# Mesenchymal stem cells: from experiment to clinic

**DOI:** 10.1186/1755-1536-4-20

**Published:** 2011-09-08

**Authors:** William R Otto, Nicholas A Wright

**Affiliations:** 1 Histopathology Laboratory, Cancer Research UK, London Research Institute, 44, Lincoln's Inn Fields, London WC2A 3LY, UK; 2 Blizard Institute, Barts and The London, Queen Mary's School of Medicine and Dentistry, 4, Newark Street, London E1 2AT, UK

## Abstract

There is currently much interest in adult mesenchymal stem cells (MSCs) and their ability to differentiate into other cell types, and to partake in the anatomy and physiology of remote organs. It is now clear these cells may be purified from several organs in the body besides bone marrow. MSCs take part in wound healing by contributing to myofibroblast and possibly fibroblast populations, and may be involved in epithelial tissue regeneration in certain organs, although this remains more controversial. In this review, we examine the ability of MSCs to modulate liver, kidney, heart and intestinal repair, and we update their opposing qualities of being less immunogenic and therefore tolerated in a transplant situation, yet being able to contribute to xenograft models of human tumour formation in other contexts. However, such observations have not been replicated in the clinic. Recent studies showing the clinical safety of MSC in several pathologies are discussed. The possible opposing powers of MSC need careful understanding and control if their clinical potential is to be realised with long-term safety for patients.

## Introduction

There has been an explosion of literature in the field of mesenchymal stem cells (MSCs) in the past 10 years. Many researchers have sought to exploit their potential as a source of reparative cells for clinical use in a variety of contexts. There are, however, several pitfalls that it would be useful to avoid, as MSCs have some awkward properties that may make their use for tissue repair or tissue engineering somewhat risky. In this review, we highlight some recent advances in the understanding of the cell biology of MSCs, and how these may integrate into strategies for more clinical applications.

Adult MSCs are generally thought of as an autologous source of reparative cells, in contrast to the totipotent, and allogeneic embryonic stem cells (ESCs). A major source of adult stem cells is the bone marrow (BM), from which two main populations derive: haematopoietic stem cells (HSCs), which produce the blood-cell lineages, and MSCs, which provide the bone-marrow stromal niche and have the potential to produce several cell lineages, including adipogenic, osteogenic and chondrogenic lineages. BM also contains endothelial precursor cells (EPCs) and probably a common precursor of all three stem-cell types, which remains somewhat less well defined. Recent reports suggest that MSCs may differentiate into endothelial cells [1,2], an outcome that may depend on the cytokine context. MSCs can also be obtained from the stromal fraction of lipoaspirates of adipose tissue [3], and these possess similar properties to BM-derived MSCs. Further sources of human MSCs include the intestinal [4], limbal [5], knee-joint [6-9] and prostate [10] stroma, trachea [11], nasal mucosa [12], Wharton's jelly (WJ) [13,14], cord blood [15] and placenta [16]. MSCs-like cells have also been extracted from tumour stroma [17], and may have an important role in the fibrotic responses, as reported for the rat kidney [18] and rat heart [19]. Thus, these cells are becoming almost ubiquitous residents in many tissues and organs. A very recent finding may even cast doubt on the utility of MSCs themselves; Medici and colleagues [20] reported that endothelial cells treated with either bone morphogenetic protein (BMP)4 or transforming growth factor (TGF)β2 reverted to a multipotent cell with some characteristics of MSCs, and could be differentiated into several endodermal cell types. The degree to which such cells or 'true' MSCs can be used in tissue repair, gene or cancer therapy may have a bearing on many clinical outcomes.

### Characteristics of MSCs from different tissues

The features of several sources of MSCs are shown in Table 1. There is considerable overlap in their gene expression patterns, as expected, but a few notable differences. The general conclusion is that MSCs or their closely related cousins can reside in or pass through most tissues, and that such cells may be isolated and/or cultured by conventional methods such as fluorescence-activated cell sorting (FACS) or plastic adherence, and can be shown to possess multipotency. It is of interest to see that MSCs also express cell-surface epidermal growth factor receptor (EGFR)-1 (Her-1, ErbB-1) and respond to the ligand heparin-binding epidermal growth factor (HB-EGF) with dose-dependent proliferation, which reversibly impairs their trilineage differentiation ability until the stimulus is removed [21]. As shown in Table 1 there is no consistent subset of surface molecules that are a definition of the MSCs phenotype; thus it is possible that many slightly different subtypes exist, and that their output phenotypes could be conditional upon local and systemic signalling.

**Table 1:** Human tissue MSC^a ^phenotypes

Tissue MSCs	Phenotypic expression		Trilineage potent
		
	Positive	Negative	
Bone marrow^b ^[118]	CD29, CD44, CD105, CD166	CD34, CD45	Yes

Umbilical-cord perivascular cells [118]	CD146, NG2, PDGF-Rb, ALP, SSEA4, Runx1, Oct4	CD34, CD45, CD144, vWF	Yes

Wharton's jelly [13,14]	CD44, CD90, CD105, HLA G6, IL-1A/B, IL-6, IL-8, IL-14, BMP1, CSF3, FAMC3, GDF15, PDGF-B, TNF-4, TNF-11b, TNF-12, VEGF	CD34, CD40, CD45, CD80, CD86	Yes

Nasal mucosa [12]	CD29, CD44, CD73, CD90, CD105, TLR4 ligand		Yes

Intestinal stroma [4]	CD29, CD44, CD73, CD105, CD117, CD166	CD14, CD34, CD45	Yes

Intestinal mucosa [4]	CD117, plus as listed for stroma		Yes

Eye limbal stroma [5]	CD105, CD106, CD54, CD166, CD90, CD29, CD71, pax-6/p75, SSEA1, Tra-1-61, Tra-1-81, CD31, CD34, CD45, CD11a, CD11c, CD14, CD138, Flk1, Flt1	Vascular endothelial cadherin	Adipo, Osteo

Osteosarcoma stroma [120]	CD44, CD73, CD90, CD105, CD166, HLA class I	CD14, CD19, CD31, CD34, CD45, HLA-DR	Yes

^a^Abbreviations: ALP, alkaline phosphatase; BMP, bone morphogenetic protein; CSF, colony-stimulating factor; GDF, growth differentiation factor; HLA, human leukocyte antigen; IL, interleukin; PDGF, platelet-derived growth factor; MSC, mesenchymal stem cell; SSEA, stage-specific embryonic antigen; TLR, Toll-like receptor; TNF, tumour necrosis factor; VEGF, vascular endothelial growth factor; vWF, von Willebrand factor.

^b^BM MSCs have been reviewed in depth many times, and interested readers are referred to several recent papers on them [28,121-125].

There are many protocols used to determine the phenotype of MSCs. The essential characteristic is their multipotentiality, which is usually established *in vitro *by retrospective analysis of their ability to differentiate into at least three cell types: typically adipocytes, osteocytes and chondrocytes, and these potencies may vary between mouse strains [22]. Other cell types may be included, depending on the purpose of the study, such as muscle or tendon cells [23]. A few researchers have used single-cell clones as a source of trilineage-potent MSCs [24], whereas others have shown their potential by *in vivo *analysis in xenografts [25].

The original characteristic of MSCs - their ability to form fibroblastic colonies *in vitro *from BM or other tissues plated onto tissue-culture plastic [26] - has been widely exploited, and there is a vast literature on their many phenotypical characteristics [27-29]. As briefly illustrated in Tables 1 and 2, there are many combinations of cell-surface markers that can be used to select MSCs from mixtures of cells, which are often performed by cell sorting using FACS [30] or immunomagnetic [31] methods, although many studies use plastic-adherent stromal cells that are subsequently classified using immunofluorescent phenotyping or flow cytometry [5,32]. The exact equivalence of these phenotypes may be questioned, but most studies have shown that the selected cells possess similar multipotentialities. A recent study [10] found by FACS analysis that some murine prostate cancer xenograft MSCs possessed a Hoechst 33342 'side population (SP)', similar to that observed more commonly for HSCs [33] and for some epithelial stem cells such as keratinocytes [34] or tumour cells in the colon [35], although perhaps not all SP cells from colon-cancer cell lines are stem cells [36]. It is possible that the murine stromal SP cells resemble those described as human adipose tissue-derived MSC- and EPC-like cells (CD34+, CD90+) that could differentiate into adipocytes and endothelial cells [37] and that may be more like multipotent adult progenitor cells (MAPCs), which show some overlaps with MSCs and mesangioblasts. However, these are essentially three cell types that differ from each other in their expression profiles, and differ again from ESCs [2,38]. It has been reported that murine MSCs and MAPCs possess similar immunomodulatory abilities *in vivo *and *in vitro *by suppressing alloreactive T-cell proliferation [39].

**Table 2:** Recent clinical use of MSCs^a ^in phase I/II trials

Patients, number	Source of MSCs	Phenotype	Culture	Cells infused	Dosage	Adverse effects	MSC survival	Follow-up	Outcome	5-year patient survival	Reference
Stroke, 16	Autologous iliac crest	> 90% SH2	DMEM, 10% FCS, Cryo	5 × 10^7 ^twice	2 doses, 2-week interval	None	ND	To 5 years	HR for MSCs = 0.344	Con 34% MSCs 72%	[114]

MI, 10	Autologous iliac crest	CD73+ CD90+ CD105+	DMEM, 10%FCS	7.5 × 10^6 ^MSCs + EPCs	1 dose	None	ND	6 months	LVEF 12% up	--	[115]

MI, 53	Allogeneic unmatched 'prochymal'	CD105+ CD166+ CD45^-^	Cryo^b^	0.5 to 5 × 10^6^/kg	1 dose, intravenous	MSCs 5 pt Placebo 7	ND	6 months	FEV1 up, LVEF up, arrhythmia down	--	[126]

ALS, 10	Autologous iliac crest	CD29+ CD44+ CD90+ CD105+ CD166+	Cambrex MSC medium 10% FCS	11.4 to 120 × 10^6^	1 dose, thoracic spine	None	ND	4 years	MSCs tolerated, SC scars	--	[116]

ALS, 19 MS, 15	Autologous iliac crest	CD29+ CD73+ CD90+ CD105+ CD166+	DMEM, 10% FCS	ALS 5 × 10^7 ^MS 6 × 10^7 ^+/- ferumoxide	1 dose, intrathecal; 1 dose, intravenous	None; CD4/25 Treg cells up	Possible	6-25 months	ALSFRS stable, EDSS better	--	[32]

Refractory Crohn's Disease, 10	Autologous iliac crest	CD73+ CD90+ CD105+	DMEM, 10% FCS, Cryo	1 to 2 × 10^6^/kg [mean wt 57 kg, range 46-113 kg	2 doses, 1-week interval	Headache (3 patients) Allergy (1 patient)	ND	14 weeks	CDAI fall (5 patients; > 70 in 3 patients);3 patients worse	--	[117]

Paediatric acute leukaemia 8	Haploid parent BM	Adherent CD phenotype ND	Cryo^b^	6 × 10^4 ^to 10^7^/kg MSCs + UCBT	1 or 2 doses, 3-week interval	None	None	6.8 years, no chronic GVHD	All patients PMN+ at 9 to 28 days	63%	[109]

Leukaemia 12^c^	HLA match opposite gender HCT	CD44+ CD73+ CD90+ CD103+	ND	ND	1 dose	ND	None	0.9-138 months	HCT success, no MSCs took	ND	[110]

^a^Abbreviations: ALS, amyotrophic lateral sclerosis; ALSFRS, ALS Functional Rating Scale; BM, bone marrow; CDAI, Crohn's disease activity index; Con, control; Cryo, cryogenic mesenchymal stem-cell preservation using dimethylsulphoxide; DMEM, Dulbecco's modified Eagle medium; EDSS, Expanded Disability Status Scale; EPC, endothelial precursor cell; FCS, foetal calf serum; FEV1, forced expiratory volume in 1 second; GVHD, graft-versus-host disease; HCT, haematopoietic cell transplant. HR, hazard ratio; LVEF, left ventricular ejection fraction; ND, not determined; PMN, polymorphonuclear leukocyte (surrogate for HSC engraftment); SC, spinal cord; UCBT, umbilical-cord blood transplant (unrelated).

^b^Osiris Therapeutics.

^c^Note this study did not infuse MSCs, but analysed cultured recipient BM MSCs after sex-mismatched BM transplant for presence of donor MSCs.

### MSCs as reparative cells

#### Liver repair

It is in the context of the liver that the relationships between MSCs, fibroblasts and stellate cells (activated as myofibroblasts) become important, because the clinical presentations of hepatic fibrosis are severe. Some of these inter-relationships have been explored elsewhere [40], and it is possible that the sources of fibrosis are several: fibroblastic, stellate cell or MSCs, or combinations of these. One less-supported hypothesis suggests that hepatocytes may undergo epithelial to mesenchymal transition (EMT) during injury and that the resolution to fibrosis or a healthy outcome depends on the reverse process (MET) [41]. Although it is certain that most fibrotic cells are mesenchymal, fibrosis need not invoke the BM; recent evidence suggests that hepatocytes in a fibrotic liver have a reduced ability to sustain a progenitor cell response, due in the main to a failure to remodel the extracellular matrix laminin, and that this failure was not affected by wild-type (WT) BM transplantation (BMT) into mice that had resistant collagen I (r/r) [42]. In another model of liver fibrosis, using bile-duct ligation, Bridle *et al. *[43] reported that rapamycin inhibited EMT, the activation of stellate cells, and the synthesis of procollagen I, cytokeratin 19 and α-smooth muscle actin (α-SMA) in treated rats. These results strongly suggest an additional role for EMT in fibrosis.

In contrast to these data, a carbon tetrachloride model of acute liver inflammation showed that many of its detrimental effects could be reversed or attenuated by infusion of sex-mismatched isogenic MSCs, but not HSCs. Thus fibrosis, infiltration of polymorphonuclear lymphocytes, synthesis of collagen I and α-SMA, and expression of inflammatory were all reduced by infusion of MSCs [44]. It is possible that these responses were partly due to the upregulation of cytoglobin expression by hepatic stellate cells, which overcomes much of the oxidative stress caused by toxic agents and at the same time inhibits the activation of those cells to become myofibroblasts [45].

In a model of sclerosing cholangitis in the Abc-b4^-/- ^mouse [46], it was reported that two components of the fibrotic response arose: one from the transformation of BM-derived cells into CD34-positive and desmin-positive fibrocytes, and one from hepatic stellate cell activation, in which α-SMA was expressed. Such BM-derived cells may be producing significant quantities of paracrine cytokines; in a model of acute rat liver injury after D-galactosamine administration, infusion of conditioned medium from cultured MSCs was able to inhibit hepatocyte apoptosis and the release of markers of liver injury, and to promote the proliferation of the parenchymal cells [47]. A further mechanism of fibroblastic activation after injury may occur via the local release of platelet-derived growth factor (PDGF), which activates local fibroblasts that in turn then express basic fibroblast growth factor and chemokine (C-X-C motif) ligand (CXCL)5, which attracts MSCs into the liver where they can differentiate into myofibroblasts [48].

#### Kidney repair

The search continues for a putative kidney stem cell, and this field has been reviewed recently [49,50], as has the general field of MSCs in kidney repair [51-55]. There are reports of both HSCs and MSCs repopulating the damaged kidney, with varying degrees of significance. For humans receiving sex-mismatched kidney transplants, Grimm and colleagues reported male stromal myofibroblasts in men receiving a female kidney graft [56]. Fang and colleagues found that male cloned BM MSCs did not contribute to the nephron regeneration of female recipient mice after mercuric chloride tubular damage [57]. Although some MSCs became resident in the damaged kidneys, the fibrotic response of kidney stromal fibroblasts following unilateral ureteric obstruction was not augmented by the infusion of sex-mismatched MSCs into transgenic mice expressing both β-galactosidase and luciferase under the control of the α2 chain promoter and enhancer of collagen I [58]. These reports contrast with those of Morigi and co-workers [59], who found that human BM MSCs were associated with decreased acute proximal tubular injury after cisplatin administration in mice. Kidney function improved, as did the lifespan of the treated mice, possibly connected with the reduced levels of apoptosis and leukocyte infiltration, and the raised tubular proliferation. A further study of female mice that received male whole BM found that tubular injury after mercuric chloride was ameliorated by erythropoietin treatment, and that up to about 4% of tubular cells in these mice possessed the Y chromosome, compared with only 1.3% of those cells in control mice [60]. This suggested that there was a small but significant engraftment of BM-derived cells into the regenerating kidney. A possible mechanism for such activity may derive from insulin-like growth factor (IGF)-1, which is secreted by MSCs. Thus, mice that received cisplatin and were infused with MSCs that were silenced for IGF-1 had enhanced kidney damage compared with mice infused with untreated MSCs [61]. These *in vitro *experiments showed that small interfering RNA or antibody directed against IGF-1 were able to inhibit the proliferation and increase the apoptosis of proximal tubular cells, lending credence to a tubular effect of MSCs in those *in vivo *studies. However, such effects may have been paracrine rather than due to the possible differentiation of MSCs into tubular cells, as implied by some other studies. This conclusion was shared by Kunter *et al*., who infused MSCs into rats with glomerulonephritis induced by anti-Thy1.1 antiserum. Arterial, but not tail-vein, injections of MSCs reduced the necrosis, improved kidney function, and increased the proliferation of mesangial cells and their expression of α-SMA, yet no incorporation of MSCs into kidney structures was seen [62]. The MSCs expressed TGF-β and vascular endothelial growth factor (VEGF), but not PDGF, in these studies.

How much kidney repair is the result of bi- (or multi-) potent parietal cells at the boundary of the Bowman's capsule and the proximal tubule is still under study. Some recent data suggest that resident stem cells, rather than BM-derived cells, play a significant part in renal repair, and involve key precursors in the Bowman's capsule and proximal tubule, which express high levels of aldehyde dehydrogenase (ALDH), CD24, CD133, keratin (KRT)7, KRT19, B-cell lymphoma (BCL)2 and vimentin [63]. A similar population of cells has been reported to respond to several inflammatory cytokines (interleukin (IL)-6, IL-8, monocyte chemotactic protein-1) by the activation of the Toll-like receptor-2 pathway, which results in their increased proliferation in kidney damage [64]. These cells may affect the mortality of patients; immunohistochemical evidence of kidneys after acute tubular necrosis suggested that patients who survived possessed more proximal tubule or parietal cells that became Ki67-positive, and expressed CD133 and paired box gene-2 [65]. These authors suggested there were other cortical and medullary stem-cell populations within the tubules that assisted kidney repair and that were also Ki67-positive after damage.

The evidence remains equivocal on the ability of MSCs to do more than act as paracrine ameliorators of kidney damage. Where evidence exists for their active differentiation into kidney tubular or interstitial cells, these effects have to date been minor. Nonetheless, the secretory activities of a few cells might have a significant local healing effect.

#### Heart repair

Myocardial infarction (MI) results in much morbidity and mortality in the human population, and methods to alleviate these problems and improve outcomes are urgently needed. Coronary-artery grafting (CAG) is commonly used to bypass diseased blood conduits. Since the publication of reports suggesting that BM-derived cells may engraft into cardiac tissue, there have been numerous attempts to exploit such a possibility, and the literature has been well reviewed recently [66,67].

We highlight a few key observations that exemplify the contradictions in the literature. That MSCs can be found in both normal and diseased hearts is no surprise. Whether they engraft and transdifferentiate into cardiomyocytes after infarcts is still a moot point. In a model of rejection, Wu and colleagues [68] showed migration of MSCs to the sites of allogeneic cardiac rejection by determining expression of transduced β-galactosidase in morphologically typical fibroblasts, and also identified a minor population of apparent myocardial cells expressing the transgene and desmin. A different approach to look at heart repair used cryo-burn injury to rat cardiac tissue, which was treated with intra-cardiac injections of cloned green fluorescent protein (GFP)-expressing MSCs. The authors did not find engraftment of MSCs into cardiomyocytes or a general neovascularisation, but they did observe reduced scarring after the MSC treatment [69]. In contrast to these results, Zhang and co-workers [70] infused cloned MSCs expressing cardiac markers into rats bearing MI, and found that these cells were associated with improved vessel density in the ischaemic ventricle and stronger ejection fraction compared with unpurified MSCs or peripheral blood monocytes. Transdifferentiation into myocytes was not reported, so these effects may have been paracrine.

Another study reported that rats with MI had increased apparent differentiation of MSCs into cardiomyocytes when they were accompanied by hyperbaric oxygen (HBO) treatment, given for 2 hours daily for 2 weeks [71]. In that study, superparamagnetic iron oxide was used to label MSCs, which were revealed by staining with Prussian blue for iron. The authors found increases in the number of positive cells within the myocardium, the thickness of the left ventricular wall, the ejection fraction and other cardiac signs, compared with rats treated with MSCs alone. Local expression of VEGF, connexin-43 and troponin-T were all increased in HBO-treated rats also, and the authors concluded that if patients were to react in a similar manner, the treatment was a potentially clinically useful adjunct to current therapies.

Two recent papers have shed light on possible mechanisms of MSC-enhanced cardiac repair. Jiang and colleagues adenovirally transduced Fischer rat MSCs with Akt and Ang-1 vectors, which resulted in protection of the MSCs from anoxia *in vitro*, and enhanced their cardiac engraftment after intramyocardial injection into MI recipients [72]. The authors found better clinical signs, such as ejection fraction, blood-vessel density and echocardiography in treated rats, and concluded that the pro-angiogenic nature of the transduced MSCs contributed significantly to these effects. A further development in the understanding of cardiac repair came from Shabbir and colleagues [73], who described evidence that a mechanism of cardiac repair involving MSCs may depend on paracrine Janus kinase (JAK) and signal transducer and activator of transcription (STAT) signalling. In their study, injection of MSCs into hamster hamstring skeletal muscles caused cardiac improvement via IL-6 secretion and JAK-STAT activation (that is, increased secretion of hepatocyte growth factor and VEGF), and these effects were reduced by the administration of the pathway inhibitor WP1066, which subsequently reversed the benefits of MSCs in the failing hamster hearts.

A tamoxifen-inducible GFP-conjugated β-galactosidase switch model of murine cardiac infarction was used to assess the contribution of exogenous cells to cardiac repair [74]. Cardiomyocytes were rendered GFP-positive by tamoxifen, and the female mice were given coronary ligations followed by infarct border injections of BM-derived lineage-negative c-kit+ cells obtained from WT male mice. After 8 weeks, the mouse hearts were harvested for histology and immunohistochemistry for GFP or β-galactosidase. MI mice had dilution of their GFP+ cardiomyocytes, which were further diluted by the c-kit+ cells, and the latter cells were positive for β-galactosidase. These results persisted when MSCs were infused into the MI mice. There was no evidence of cell fusion between MSCs and myocytes. The authors concluded that MSCs do not partake in direct healing of the myocardium in this model, but rather that a significant proportion of new myocytes derive from the BM c-kit+ population, even when exogenous SDF1 was used to stimulate angiogenesis.

Such results have encouraged clinical applications of MSCs, as noted above (Table 2). Chin and co-workers [75] used intramyocardial injections of cryopreserved autologous MSCs at the time of CAG, and they reported better heart function, less scarring, improved wall thickness and fewer arrythmias than with CAG alone.

#### Colon repair

Recent work has suggested that MSCs in the colonic environment can aid or hinder tissue repair. Thus, on the positive side, Tanaka and colleagues [76] used cultured MSCs, which were injected via the tail vein into male Lewis rats that had been treated with 4% dextran sodium sulphate (DSS); they found that the MSCs ameliorated the colitis by exerting an anti-inflammatory effect. This was in part assayed by ELISA for tumour necrosis factor (TNF)-α after co-culture of lipopolysaccharide-stimulated MSCs and a monocyte cell line. Several colonic inflammatory cytokine mRNAs and other mRNAs, such as *Tnf-α*, *Il-1β *and *cyclooxygenase 2*, were reduced by MSC infusions. Unfortunately no *in situ *hybridisation was performed to confirm which cells were involved in these reactions. Nevertheless, the overall effects of the MSCs on the clinical and biochemical effects of DSS were positive. In a similar DSS colitis model, Yabana *et al *[77] infused male enhanced (e)GFP-MSCs into female rats treated with the BM hypoplastic reagent busulphan. The colitis was worse in the BM-depleted animals, and in large part rescued by MSCs, which strongly stimulated induction of synthesis of tight junction proteins to alleviate the loss of barrier function in the epithelium. These authors found occasional Y-chromosome-positive CD45-negative cells within the epithelial monolayer of rats treated with DSS and MSCs, suggesting that some MSC-like cells might have entered this compartment, but it was not certain which phenotype these cells possessed.

Similarly, Grisendi *et al *[78] used adipose tissue-derived MSCs that were transduced with TNF-related apoptosis-inducing ligand (TRAIL) to target several xenograft models of cancer, including cervical, pancreatic and colon cancer. Raised levels of apoptosis were found in all cases, in particular in TRAIL-resistant breast-cancer cells. Cancer cell caspase 8 activity was raised, with little apparent normal tissue toxicity. These results suggest that it could be possible to direct MSCs therapeutically towards tumour cells.

By contrast, Wu and co-workers reported a model of fibromatosis (desmoid tumour) in the intestines of the mutant Adenomatous polyposis coli (*Apc*^*Min*^) mouse [79]. The number of intestinal desmoids was proportional to the number of MSCs present, and the MSCs were held in a less differentiated state than in WT mice. The authors found that when Sca1^-/- ^mice, which have fewer MSCs, were crossed with *Apc^Min ^*mice, the resulting offspring developed fewer desmoids, but there was no effect on the number or severity of the epithelial polyps. The authors concluded that β-catenin has local effects on both the epithelium and the MSC populations, and maintains MSCs in an immature state, which promotes the aggressive fibromatosis. Such an outcome would not augur well for the clinical use of MSCs if human colon adult progenitor cell status is similar.

### Fibroblastic differentiation

Lee and colleagues [80] reported that human MSCs could differentiate into stromal fibroblasts *in vitro *after stimulation by connective tissue growth factor, during which they secreted collagen I and tenascin-C. MSCs were initially α-SMA-negative, but could express this protein on stimulation with TGFβ. Sarraf and co-workers [24] reported that murine MSCs could differentiate into fibroblasts and myofibroblasts when embedded in a collagen type I matrix and placed under tension, either self-generated or externally applied. The cells secreted both collagenous and elastic fibres. It thus seems likely that BM-derived myofibroblasts and fibroblasts in unsorted BMT experiments have come from the MSC population.

Several publications have reported a flux of BM-derived stromal myofibroblasts and fibroblasts into many tissues [81,82], and that damage increases their numbers. Direkze and colleagues [82] found that lung tissue damaged by paracetamol contained 41% BM cells, compared with 17% in control lungs. A similar pattern was found in full-thickness wounded skin, but with only 4% of BM-derived myofibroblasts. Interestingly, the proportion of kidney-derived myofibroblasts did not rise significantly with injury, but remained at around 20 to 24%. This group also reported BM-derived tumour-associated myofibroblasts and fibroblasts in a mouse model of insulinoma [83].

Zhao and colleagues reported that the marker CD90 was raised in both MSCs and in prostate cancer stromal fibroblasts, which the authors analysed by quantitative PCR after isolating them from tissues by FACS, using CD90 as a discriminator [30]. The authors concluded that the CD90-hi cells were not true MSCs, but that they expressed several proteins associated with tumour promotion, such as Sonic hedgehog and TGF-β, as well as pro-angiogenic factors. A further report on lung MSCs suggested that these also respond to TGFβ by differentiating into myofibroblasts [11].

### Variability between MSCs

#### Immunogenicity

In general MSCs are thought to be immunosuppressive, with some impressive reports supporting this [14], but there are also reports that suggest that this may not always apply [84]. On the positive side, an animal study of BM engraftment compared syngeneic and allogeneic MSCs with allogeneic fibroblasts in a model of excisional skin wounding. The allogeneic fibroblasts set up a CD45, CD3 and CD8-positive leukocytic response that was far greater than those to either of the MSC sources, both of which healed wounds faster.

Human MSCs obtained from WJ were found to be somewhat benign in their effects [14]: lack of splenocyte proliferation in an allogeneic mixed lymphocyte reaction after Concanavalin A stimulus; lack of surface expression of the co-stimulatory molecules CD40, CD80 and CD86 and of the expression of IL-6 and VEGF, generally held to be immunomodulatory when found on MSCs. The authors concluded that this source of MSCs was a possible option for allogeneic transplantation.

Hsieh and colleagues [13] reported gene-array comparisons using BM MSCs and WJ MSCs. The BM MSCs showed upregulation of antigen-presentation genes and genes of cytokine pathways; WJ MSCs expressed more neurogenic, angiogenic, proliferation and nuclear factor kappa B-related genes. Multidimensional scaling analysis suggested a closer relationship of WJ cells to ESC, whereas BM MSCs were more related to fibroblasts and adipocytes. It was also noted that the gene profiles of BM MSCs were more sensitive to the culture growth media, whereas the genes expressed by WJ MSCs were more similar in the two media tested (either MesenCult or Dulbecco's modified Eagle's medium with 10% FCS). The authors concluded that WJ cells may constitute cells that are less likely to be rejected and more versatile in their differentiation potential than BM MSCs.

In contrast to the above results, Sbano and colleagues reported on an allogeneic skin-transplant model in which Sprague-Dawley rats were immunosuppressed with cyclosporin A (CsA) and given Wistar donor MSCs [85]. The allogeneic MSCs promoted skin rejection in the non-immunosuppressed rats compared with the CsA-treated rats, and this was associated with higher levels of inflammatory cytokines such as IL-2, TNF-α and interferon (IFN)-β.

#### Tumour promotion

Houghton and co-workers [86] reported that MSCs transfected with mutant p53 and injected into *Apc^Min ^*mice resulted in enhanced mammary tumour load, in contrast with WT mice, from which live cells could be harvested up to 1 year later from the BM, and in which no tumours occurred. The mutant p53 MSCs homed to the breast stroma, and were permissive for carcinoma development. These tumours were also responsive to exogenous anti-TNF-α treatment or to the infusion of regulatory CD4 T cells obtained from WT donors.

A report from Johann and colleagues [17] suggested that paediatric tumour stromal cells may possess similar immunophenotypical and lineage properties to BM-derived MSCs, and might also suppress proliferation of peripheral blood monocytes *in vitro*, as well as the cytotoxicity of natural killer cells in co-culture assays. It was suggested that these properties of the tumour stroma enhance the ability of a tumour population to evade immune surveillance, with augmented patient risk.

Maas and colleagues reported recently [87] that the protein Artemis is crucial in the repair of double-strand breaks in the DNA of MSCs through the non-homologous end-joining pathway (NHEJ), and that if knocked out, it may predispose MSCs to sarcoma formation. The authors tested this theory in p53-delta/+ heterozygote mice cross-bred with Artemis-null mice, and found that 6% of the mice developed sarcomas in cartilage, bone and skeletal muscle. MSCs from Artemis-deficient mice exhibited more frequent double-strand breaks, translocations and fragmentations than did their WT littermates, yet retained a normal growth rate and ability to differentiate along the usual trilineage pathways. The radiosensitivity of Artemis-null and WT cultured MSCs was greater than that of mouse embryo fibroblasts up to 5 Gy, and there were significant changes in the expression of the BMP and Wnt pathway, stress-response and proliferation/differentiation genes in the defective MSCs. Unfortunately, the authors did not show that the sarcomas in their mice were MSCs-derived. Nevertheless, these alterations led to the conclusion that MSCs need careful monitoring for genetic stability, particularly of DNA checkpoint machinery, if they are to be useful in regenerative medicine.

A study using a model of human prostate cancer, PAC-120 xenografting [10], suggested that there is an SP (Hoechst 33258-pumping) of murine host stromal cells that can be sorted by flow cytometry and differentiated *in vitro *into adipocytic and osteocytic lineages. The cloned SP cells had characteristics of MSCs, as shown further by their positivity for Sca-1 and CD81. A problem with these results is that no immunohistochemistry was used to show the presence of MSC cells within the stroma of the tumours. The cells obtained by flow cytometry of crude enzymatic digests of the xenografts were by definition heterogeneous, and the authors identified a broad SP that was not well controlled by verapamil. The sorted MSC-like cells may have been closely associated with the epithelial fractions being subjected to the SP sorting, so although it seems true to suggest that the stromal fraction contained MSC-like cells, it is unusual for such cells to possess an SP, unlike their BM-derived neighbours, the HSCs.

### Xenotransplantation

MSCs are common to all mammals tested to date, and have been viewed as essentially benign because of their frequent lack or suppression of immune effects on hosts. However, an increasing body of literature has reported unfortunate or even malign effects that may result from xenogeneic MSC infusions.

Early work on MSC infusions used human MSCs in foetal sheep [25,88], which suggested that this immune-privileged site is permissive for tolerance of foreign cells, which can engraft, differentiate *in situ*, and function in a normal way for that cell type for extended periods; in those studies, up to a year. MSCs were detected in cartilage, fat, muscle, heart, BM stroma and thymic stroma.

Much of the clinical literature to date on infusion or injection of MSCs in human disease has failed to show significant adverse effects (AEs) on the recipients [89], particularly for their use in HSC transplantation [90]. This is encouraging for possible therapeutic uses of MSCs in tissue repair and regenerative medicine. However, there are reports of human MSCs in xenograft models of disease suggesting that some caution is needed regarding the absolute benevolence of these cells. These reports may reflect differences in the cell biology of the species involved, and need not predict that the same pathologies will occur in people, but nevertheless indicate that the precautionary principle should apply. Some recent examples are presented below.

Teng and colleagues reported that human MSCs could be rendered tumourigenic by the hypermethylation silencing of two tumour suppressor genes that activate p53: Hypermethylated in cancer (HIC)1 and RassF1A [91]. These cells were transformed, grew as anchorage-independent colonies in agar, and formed sarcoma-like tumours when injected subcutaneously into nude mice.

Several reports now suggest a strong possibility that MSCs may be permissive for the proliferation and dissemination of breast-cancer stem cells. For example, Yan and colleagues extracted MSCs from breast tumours that possessed trilineage potential and augmented the growth of mammary tumours when co-infused into animals. The MSCs also stimulated 'mammosphere' formation *in vitro*, which was EGF-dependent [92]. Similar effects on mammosphere formation were found by Klopp *et al *[93], who found reduced E-cadherin expression in normal and breast-cancer epithelial cells, and the MSCs augmented tumour development when co-injected into mice.

It may be that MSCs can differentiate into fibroblastic cells by the action of breast tumour-secreted osteopontin, and by so doing enhance the tumourigenic and metastatic potential of the MDA-MB231 cancer cell ine [94]. Human MSC cultures possess a subpopulation of ALDH-positive cells that stimulate the induction of cancer stem cells (also ALDH+) in the human breast line SUM159 *in vitro *[95]. When non-obese diabetic/severe combined immunodeficiency (NOD/SCID) mice bearing such tumour xenografts were given intratibial injections of human MSCs, the MSCs homed to the tumours, which grew faster and possessed more cancer stem cells than did control tumours. It was inferred from microarray analyses of tumour cell-MSC co-cultures that several cytokines may be expressed in the tumour cells that act to promote these effects: CXCL1, CXCL5 and CXCL6, IL-6 and IL-8. Antibodies to CXCL7, itself a product of MSCs, blocked the secretion of these cytokines. The regulation was further controlled by breast-tumour cell-derived IL-6 stimulating both the secretion of CXCR7 and the chemotaxis of MSCs to the tumour cells. The MSCs then increased the population of ALDH+ epithelial cancer stem cells. These authors also reported similar juxtapositions of ALDH+ MSCs and breast-cancer cells in sections of human breast tumours.

In a similar study, Zimmerlin and colleagues studied adipose-derived MSCs, and reported the ability of these cells to enhance the growth of breast-tumour pleural effusions from patients in both *in vitro *and *in vivo *settings [96]. The authors separated the metastatic breast-tumour cells into active and resting phenotypes based on CD90 positivity, with high or low scatter respectively. When co-injected with the adipose-derived MSCs into mice, only the active effusion cells were tumourigenic.

Park and co-workers reported the migration of human umbilical-cord MSCs towards human glioma cells *in vitro*, and that overexpression of CXCR4 increased this trait. Further, in a xenograft model of glioma in nude mice, these cells displayed enhanced migration into the tumours [97]. In an experiment in mice using transplantation of GFP-tagged BM, GFP-positive MSCs migrated into the prostate of castrated mice, and these cells were increased by testosterone in a Wnt-dependent manner. These findings were also seen in a human prostate tumour xenograft, in which MSCs expressing an exogenous Wnt antagonist, secreted Frizzled-related protein (SFRP)-2, induced tumour shrinkage by necrosis and apoptosis [98].

Kucerova and colleagues reported that adipose-tissue MSCs could promote growth in nude mice of tumours of the xenografted human melanoma cell line A375 [99]. This was achieved by suppression of apoptosis and an increase in proliferation. Another melanoma line, 8MGBA, did not share this attribute; instead, MSCs were inhibitory.

Two recent reports suggest that MSCs may give rise directly to mesenchymal tumours. Using a comparison of infused normal MSCs, *in vitro *spontaneously transformed MSCs, and osteosarcoma murine cells, Mohseny and co-workers [100] concluded that aneuploidy, chromosomal translocations and the homozygous loss of the Cdkn2A (p16) locus on chromosome 4 were implicated in tumour progression. The genetic changes seemed to occur around MSC passages 5 to 9 in culture, during which time the cells went into 'crisis', and thereafter they possessed the ability to grow in soft agar independently of substrate. The authors showed a series of 88 human osteosarcomas that possessed similar defects in the homologous cyclin-dependent kinase inhibitor (CDKN)2A locus on chromosome 9. Kaplan-Meier analyses of these patients with osteosarcoma showed very poor survival for patients negative for this locus (zero survival at 50 months follow-up versus 70% survival if positive for more than one allele). Although this study did formally prove the origins of these human osteosarcomas to be MSCs, it warrants a cautious approach when using these cells in the clinic.

A further report of tumours arising from genetically defective MSCs has recently appeared [101]. These authors deleted p53, Rb or both genes in adipose tissue-derived murine MSCs that underwent Cre-LoxP excision. Wild-type and Rb-negative MSCs were phenotypically normal, whereas the p53-negative and p53-negative/Rb-negative MSCs were transformed, and could initiate leiomyosarcomas in half the animals when injected into the flanks of NOD-SCID/IL-2Rγ^-/- ^mice. The transformed MSCs approached tetraploidy, and were deficient in the ability to differentiate into adipocytes, yet had increased ability to become osteocytic. The authors noted that human leiomyosarcomas frequently display loss of p53 or Rb.

These examples indicate the possibility that MSCs could be involved in the growth of carcinomas, melanomas and sarcomas, and thus their use as repair agents for normal tissues or organs needs to be seen in this light. In addition, MSCs may also be exploited precisely for their homing attribute; by modifying them appropriately, infused MSCs may home in on tumours and deliver therapeutic reagents. Such experiments have been reported for an anti-tumour viral vector Delta-24-RGD transfected into MSCs, which homed to breast and ovarian tumours in mice and reduced systemic viral toxicity to negligible levels compared with virus-alone infusions [102]. A different strategy was used by Sato and colleagues, who transfected MSCs with EGFR; these cells homed to both B16 murine melanoma and GL261 glioma tumours [103]. When the MSCs were co-transfected with IFN-α, there was significantly increased survival of GL261-bearing mice. Secchiero and colleagues [104] reported recently that BM MSCs could affect the outcome of Epstein-Barr virus (EBV)-positive or EBV-negative metastatic non-Hodgkin's lymphomas in nude-SCID mice. Mice receiving MSCs survived for longer periods than those without (40 and 59 d median survival, respectively).

Human MSCs have been used as a model for tumour therapy after transduction with IFN-β [105]. Two murine pulmonary metastatic xenograft models were used: A375SM melanoma and MDA 231 breast carcinoma. In both models, the IFN-β MSC infusions resulted in MSC engraftment within the tumour stroma, and significantly prolonged survival of the mice compared with IFN-β injections alone. These results encourage research into the personalising of such treatments for suitable patients.

### Clinical use of MSCs

Clinical trials using MSCs are being carried out for a variety of important diseases such as stroke, MI, multiple sclerosis (MS), amyotrophic lateral sclerosis (ALS), and leukaemia (Table 2). In general, MSCs appear to be well-tolerated, with most trials reporting lack of AEs in the medium term, although a few showed mild and transient peri-injection effects. There are no agreed phenotypic MSC markers that should be used, so the exact clinical effects of such sorted cells may be uncertain, as the cell populations used could differ. In addition, clinical outcomes are variable, and generally show small improvements, but to date few studies have reported either a long period of observation, the outcomes of more than one MSC infusion, or whether MSCs survive engraftment. That human MSCs circulate in the bloodstream has been reported [106,107], and immortalized MSC lines have been produced from similar cells [108]. There may be a direct effect of the infused cells, but long-term clinical MSC engraftment is not yet routinely investigated by methods such as paramagnetic iron nanoparticles. Where this test has been performed, it has shown only possible survival of MSCs [32], or none at all [109]. It was found that the BM engraftment of donor MSCs in patients with leukaemia receiving whole-body irradiation and HSC rescue did not occur, but this did not influence the HSC engraftment [110,111]. However, those data are in contrast to studies in which sex-mismatched BMT resulted in donor-derived stromal cells in several organs including liver [112], and endothelial cells in the BM of patients with chronic myeloid leukaemia [113]. It is possible that some major influences of transplanted MSCs are systemic or paracrine via the release of cytokines or other molecules that affect responses in the target organ. Such influences are exemplified by a recent study on a rat model of hepatic failure, in which anti-apoptotic effects were seen after infusion of cultured MSC-conditioned medium [47].

Autologous MSC infusions were performed in 16 patients with severe middle cerebral artery stroke, who were successfully followed up for up to 5 years, during which 58% of controls but only 25% of MSC-infused patients died [114]. All patient MSCs were cultured in the presence of 10% FCS, and harvested to achieve 10^8 ^cells/person, then delivered in two intravenous infusions of 5 × 10^7 ^cells, 2 weeks apart. No side effects were noted, and similar levels of other disease parameters (vascular problem, seizure) were seen in both groups of patients. There was an association between MSC infusion and the levels of serum stromal cell-derived factor-1.

In a phase I trial, Lasala and co-workers [115] infused a mixture of fresh peripheral mononuclear cells (a source of EPCs) and cultured BM MSCs (7.5 × 10^6 ^each) into the ischaemic myocardium of patients with angina pectoris who had over 70% stenosis in one or both coronary arteries. Left ventricular ejection fraction was increased by 12% at 1 month, and remained with an 11% increase at 6 months after the infusions, and cardiac ischaemia was decreased by 1.8-fold at 6 months only. The patients reported increased quality of life, and no AEs were seen. This is encouraging because the MSCs were cultured in bovine serum during their expansion *in vitro*.

Another phase I trial used cultured autologous MSCs in patients with ALS [116]. The cells were infused in cerebrospinal fluid into the thoracic spinal canal, and patients were monitored by MRI for 4 years, during which no AEs were noted locally or systemically. No attempt was made to track the survival of the MSCs. There was little change in the disease progression.

In a similar study, Karusis *et al *[32] studied patients with MS and patients with ALS, who also had no AEs after a single intrathecal infusion of autologous MSCs. In some patients, the MSCs were labelled with superparamagnetic iron-oxide nanoparticles, and there was evidence of their retention in the occipital horns of the ventricles, the meninges of the spinal cord, the parenchyma and the nerve roots for up to 3 months. In short-term (24 hours) immunological analyses of circulating blood cells, there were 72% more regulator T cells (CD4/CD25+) in both patient groups, and reductions of 30 to 60% in the proportions of myeloid dendritic cells positive for CD83, CD86 and human leukocyte antigen (HLA)-DR. Phytohaemagglutinin-stimulated lymphocytes were also 63% less reactive than before infusions. These data strongly suggest a decrease in the activation status of the host lymphocytic cells and antigen-presenting cells after MSC treatment.

A phase I trial of autologous MSCs (1 to 2 × 10^6 ^cells/kg twice) for refractory Crohn's disease (CD) has been reported [117]. CD MSCs were similar to normal MSCs in immunomodulatory effects and phenotype, and did not cause side effects. Five common drugs used in the treatment of CD (adalimumab, methotrexate, azathioprine, dexamethasone, 6-mercaptopurine) all allowed MSCs to inhibit the proliferation of peripheral bone-marrow cells in *in vitro *tests, whereas infliximab had a similar but non-significant trend. Three of the ten patients improved their Crohn's Disease Activity Index, but three were worse by 6 weeks after treatment, and required surgery. The authors concluded that the methods were safe, but warranted further longer-term investigations.

A report was recently published which detailed a study involving patients with paediatric leukaemia who received a transplant of unmatched umbilical-cord blood with parental haploidentical BM-derived cultured MSCs on one or two occasions [109]. Any graft-versus-host disease (GVHD) reaction that occurred was acute and responded to steroid therapy, and no episodes of chronic GVHD were noted in the 6.8 year study. Using blood analyses, all patients were found to be chimaeric with regard to all HSC-derived blood-cell lineages within 3 months of transplant (positive for polymorphonuclear leukocytes by 9 to 28 days and platelets by 36 to 98 days), which was comparable with historical control patients who had received no MSC treatment. All patients were analysed by BM sampling for surviving donor MSCs and the degree of BM white blood-cell chimaerism at varying times after engraftment. At no time were any donor MSCs found, despite full haematopoietic chimaerism. This is of considerable interest because many of the putative benefits of MSCs treatment presume long-term engraftment of the cells, which may not have occurred here. It remains possible that some MSCs had engrafted into unsampled organs and exerted a systemic effect from those locations. This possibility would apply to another leukaemia study [110], in which 12 patients with leukaemia were given sex-mismatched but HLA-matched BMT, and their BM was analysed up to 11 years later for evidence of donor-derived MSCs. In all cases, the BMT resulted in full blood-cell chimaerism, but in no case was there any evidence of donor BM MSC survival. This was true for each of three different conditioning regimens before BMT. Again, other body sites were unsampled. It is possible that the haplotyping was not sufficiently close to prevent a host response against the infused MSCs.

## Conclusions

Over the past decade, there have been a large number of publications on MSCs from several tissue sources. A number of animal models of human diseases have shown encouraging results for the use of MSCs in terms of repair and restoration of functional tissue, as have a fewer but growing number of human studies. The proportion of transplanted cells taking part in these repairs remains low, and it is unclear how many cells survive the grafting events and what proportion of their effects are due to systemic signalling or direct cell-cell communication.

We urge caution about use of MSCs, for several reasons. Xenotransplantation of animal MSCs seems inherently unattractive from the recipient's viewpoint, not least for the possibility both of rejection and of viral or other disease transmission. Use of allogeneic human MSCs may have some utility if it can be unequivocally shown that no possibility exists for tumour growth in the longer term, either of the MSCs themselves (possibly as transdifferentiated sarcomas), or by enhancing the growth of epithelial tissues prone to such events by the stimulation of blood vessel ingrowth. These strictures will also apply to autologous MSCs for the specific repair of certain conditions. Use of MSCs in patients with cardiac infarcts could be helpful as adjuncts to CAG, and it is likely that their use will progressively expand into several other disease states as safety profiles improve, and the sourcing and purification or culture methods become less expensive, allowing more routine use.

## List of Abbreviations

ALS: amyotrophic lateral sclerosis; BM: bone marrow; BMP bone morphogenetic protein; CAG: coronary artery grafting; CDAI: Crohn's Disease Activity Index; Cryo: cryogenic mesenchymal stem-cell preservation using dimethylsulphoxide; DMEM: Dulbecco's modified Eagle medium; DSS: dextran sodium sulphate; ELISA: enzyme-linked immunosorbent assay; EMT: epithelial to mesenchymal transition; EPC: endothelial progenitor cell; ESC: embryonic stem cell; FACS: fluorescence-activated cell sorting; FCS: foetal calf serum; GFP: green fluorescent protein; GVHD: graft-versus-host disease; HBO: hyperbaric oxygen; HSC: haematopoietic stem cell; MET: mesenchymal to epithelial transition; MI: myocardial infarction; MSC: mesenchymal stem-cell; NHEJ: non-homologous end-joining; WJ: Wharton's jelly; WT: wild type.

## Competing interests

The authors declare that they have no competing interests.

## Authors' contributions

WRO researched and wrote the draft. Both authors discussed and corrected the manuscript.

## Authors' information

WRO has worked on MSC and BM cell plasticity as well as trefoil peptide biology for over 20 years. NAW has led a research group at Cancer Research UK for over 20 years and worked on a variety of topics including the origin and clonal spread of epithelial cancers, particularly in the gastrointestinal (GI) tract and other visceral organs, the ability of BM cells to contribute to cell population dynamics and functions of other organs, and the roles of trefoil peptides in GI disease.
